# Transformer-based NLP approaches for credit risk prediction: a systematic review

**DOI:** 10.3389/frai.2026.1819994

**Published:** 2026-05-15

**Authors:** Pfarelo Raliphada, Seun Olukanmi, Micheal Olusanya

**Affiliations:** 1 School of Computer Science and Applied Mathematics, University of the Witwatersrand, Johannesburg, Gauteng, South Africa; 2 Department of Computer Science and Information Technology, Sol Plaatje University, Kimberley, Northern Cape, South Africa

**Keywords:** BERT, classification, credit risk, LLaMA, Natural Language Processing (NLP), RoBERTa, sentiment analysis, transformer models

## Abstract

**Introduction:**

This study systematically reviews transformer based Natural Language Processing (NLP) and Large Language Model (LLM) approaches for credit risk prediction, addressing limitations of traditional structured data credit scoring models.

**Methods:**

A PRISMA guided systematic literature review was conducted across Scopus, ScienceDirect, and Web of Science, covering English language studies published between 2015 and 2025. A total of 284 records were identified and screened using semantic similarity filtering, resulting in 63 eligible studies for qualitative synthesis.

**Results:**

The findings show that transformer based architectures, including Bidirectional Encoder Representations from Transformers (BERT), Robustly Optimized BERT Pretraining Approach (RoBERTa), and Large Language Model Meta AI (LLaMA), consistently outperform traditional statistical and machine learning baselines across financial prediction tasks. Attention based Long Short Term Memory (LSTM) models reported Area Under the Curve (AUC) improvements of 3.08% and KS increases of 10.3% over classical methods, while hybrid Convolutional Neural Network (CNN) Transformer architectures achieved accuracy levels up to 96.9% and F1 scores of 0.995 in credit risk datasets. Multimodal and transformer based systems also reported accuracy levels exceeding 95% in financial risk monitoring tasks. However, only a limited subset of studies incorporates formal explainability frameworks or fairness evaluations.

**Discussion:**

Transformer based NLP approaches improve credit risk prediction by effectively leveraging unstructured textual data. Nevertheless, challenges remain in interpretability, transparency, regulatory alignment, and ethical deployment. Future research should prioritize bias mitigation and governance aware model design to support responsible use in regulated financial environments.

## 1 Introduction

The primary focus of financial institutions is to assess and manage credit risk, necessitating the development and enhancement of complex credit risk models, as highlighted by (Pathak et al. 2023). This literature review provides a comprehensive overviesw of how transformer-based Natural Language Processing (NLP) and Large Language Models (LLMs) are used for credit risk classification, highlighting key studies and developments in the field. Accurate credit risk classification is critical to ensure the stability and profitability of financial institutions within the broader scope of financial risk management, as indicated by (Lessmann et al. 2015), that demonstrate that traditionally, credit scoring systems have relied largely on structured data and statistical methods to evaluate a borrower's credibility. However, due to the rapid increase in unstructured financial text data, such as loan descriptions, transaction records, and customer communications, there is a growing recognition of the importance of utilizing these data to improve credit risk evaluations, as noted by (Wang et al. 2019).

Natural Language Processing (NLP) models, particularly transformer-based architectures such as Bidirectional Encoder Representations from Transformers (BERT), Robustly Optimized BERT Pretraining Approach (RoBERTa), and Large Language Model Meta AI (LLaMA), have enabled financial institutions to derive actionable insights from unstructured textual information. These models have shown significant improvements in classification tasks by capturing syntactic and semantic nuances in textual data, thus increasing the predictive accuracy of traditional credit risk models, as demonstrated by (Jin and Zhang 2024). For instance, attention-based LSTM networks have been used to analyze borrower behavior patterns in peer-to-peer (P2P) lending platforms, resulting in enhanced credit scoring performance, as shown (Jin and Zhang 2024).

Studies have extended the application of transformer-based architectures, originally developed for NLP, to other domains such as healthcare, specifically for Alzheimer's disease risk assessment from speech transcripts, demonstrating their effectiveness in early disease detection (Roshanzamir et al., 2021). While these studies are not directly related to credit risk, they are included to illustrate transferable methodologies that can be adapted to financial risk modeling. By leveraging models such as Bidirectional Encoder Representations from Transformers (BERT), researchers have demonstrated the efficacy of NLP in extracting meaningful risk indicators from loan narratives, as shown by (Sanz-Guerrero and Arroyo 2025). The integration of temporal models, including Temporal Convolutional Networks (TCN) and DilateFormer, with NLP techniques further refines classification accuracy by capturing temporal dependencies alongside textual semantics, as demonstrated in recent studies (Shen and Wu, 2025).

Despite these enhancements, several challenges remain. The interpretability of deep learning models, concerns regarding privacy, and the integration of insights derived from NLP into regulatory-compliant frameworks continue to impede the widespread adoption of these approaches, as highlighted by (Liu et al. 2020). Furthermore, the current literature lacks a cohesive methodology for evaluating and benchmarking NLP-based credit risk classification models, leading to a fragmented understanding of their comparative effectiveness, as noted by (Lessmann et al. 2015).

These enhancements and ongoing challenges highlight the evolving role of NLP in credit risk modeling within financial analytics. Research emphasizes how large language models and transformer architectures can enhance traditional credit scoring systems. However, to fully utilize these benefits, it is necessary to address issues related to model transparency, data governance, and standardized evaluation. Overcoming these challenges is critical for the effective and ethical use of NLP in credit risk classification in real-world finance, as emphasized by (Pathak et al. 2023) and (Heng and Subramanian 2022).

This systematic literature review aims to analyze how NLP techniques have transformed credit risk model classification. It evaluates how these advancements enhance risk management, improve credit scoring accuracy, and support informed decision-making in financial institutions. The focus is on the application of transformer-based NLP models in credit risk classification and the role of unstructured data in improving predictive accuracy. Additionally, the review discusses limitations, ethical challenges, and interpretability issues related to NLP in credit risk modeling, highlighting the need for responsible deployment within a regulated industry.

This literature review is guided by several core research questions:

- RQ1: How have NLP techniques, particularly advanced transformer-based models such as BERT, RoBERTa, and LLaMA, been applied to improve credit risk classification?
- RQ2: What benefits and challenges arise from incorporating unstructured textual data, such as customer communications, loan descriptions, and market sentiment, into credit risk models through NLP methodologies?
- RQ3: Which NLP-based approaches or hybrid models demonstrate the greatest potential for enhancing credit risk model performance, and how do they compare with traditional and non-transformer machine learning methods?
- RQ4: What limitations persist in the application of NLP-based credit risk models, particularly with respect to model transparency, predictive reliability, ethical considerations, and regulatory compliance?

In contrast to previous reviews that address the broader themes of machine learning or explainable artificial intelligence within the context of credit risk, this study presents the inaugural systematic synthesis centered on transformer architectures in NLP for credit risk modeling. This analysis integrates semantic filtering techniques, offers a comparative taxonomy of existing models, and includes a thorough examination of the ethical and regulatory considerations associated with these methodologies. In this study, a PRISMA-guided systematic literature review is conducted to identify, evaluate, and synthesize existing research on transformer-based NLP approaches for credit risk prediction. The methodology integrates structured database querying with semantic similarity filtering to ensure that only thematically relevant studies are included. A total of 284 records were initially identified across Scopus, ScienceDirect, and Web of Science, and following deduplication and filtering procedures, 63 studies were selected for detailed qualitative analysis. The study further categorizes the selected literature into key methodological groups, including transformer-based models, hybrid architectures, and multimodal approaches, enabling a comparative evaluation of their performance and applicability in financial risk modeling. In addition, the analysis examines the extent to which these models address critical issues such as interpretability, fairness, and regulatory compliance. By combining systematic selection, structured synthesis, and critical evaluation, this study provides a comprehensive overview of current advancements while identifying key gaps and directions for future research in credit risk modeling.

## 2 Literature review

This section provides background information on modern deep learning-based NLP models, specifically Bidirectional Encoder Representations from Transformers (BERT), Robustly Optimized BERT Pretraining Approach (RoBERTa), and Large Language Model Meta AI (LLaMA) 3.2 by (Devlin et al. 2018), (Liu et al. 2019), and (Touvron et al. 2023), within the specialized domain of credit risk classification. To maintain thematic coherence, we narrowed our review to studies that directly applied NLP and LLMs to financial and credit risk contexts (Wang et al., 2019; Sanz-Guerrero and Arroyo, 2025). To systematically summarise the reviewed studies and identify research gaps, Table 1 presents a synthesis of articles, their contributions, and identified limitations. Articles that extended into unrelated NLP classification tasks were excluded to sharpen the review's focus. Some studies from adjacent domains are included to illustrate transferable NLP methodologies applicable to financial risk modeling. We began by outlining essential NLP principles and elucidate how these sophisticated models effectively process text data, particularly in the context of financial analysis.

**Table 1:** Summary of articles, contributions, and identified gaps.

Articles	Relation between articles	Contributions	Gaps
(Xu et al. 2025)	Proposes a new end-to-end framework for clinical information extraction (NCIE) from Chinese radiology reports.	Presents the NCIE method for structured data extraction.	Dataset mainly uses chest X-ray and CT reports, limiting broader applicability.
(Lin and Liao 2024)	Introduces a lexicon-based prompting method for financial sentiment analysis.	Proposes dictionary-driven prompt integration in language models.	Limited to polarity classification; neutral or nuanced sentiment not covered.
(Du et al. 2025)	Develops a text-based ESG risk metric using Word2vec-derived lexicons.	Quantifies ESG risks from earnings call transcripts using text analytics.	Limited focus on direct ESG risk quantification in prior literature.
(Corradi et al. 2022)	Applies NLP for mechanistic information extraction in toxicology.	Demonstrates NLP support for structured toxicological information extraction.	Requires automation to unify biological events and ontology linking.
(Hu et al. 2024)	Presents a BERT-IDCNN-CRF model for entity extraction in R&D abstracts.	Introduces hybrid deep architecture for scientific entity recognition.	Dataset annotation complexity limits generalization.
(Babaalla et al. 2024)	Comparative analysis of UML diagram extraction from natural language specifications.	Evaluates accuracy of NLP and ML-based UML extraction systems.	Persistent shortcomings in translating requirements to UML diagrams.
(Xu and Li 2021)	Evaluates ML algorithms for valuation and financial risk assessment for housing transactions.	Improves valuation accuracy and outperforms linear and econometric models.	Fail to capture immediate policy shocks by relying on historical data.
(Chagnon et al. 2024)	Introduces BERTeley for scientific topic modeling.	Enhances topic coherence using scientific language models.	Formatting constraints may dilute contextual learnings.

Credit risk classification involves evaluating the likelihood of default by individuals or entities based on their historical performance and contextual information. The complexity of this undertaking lies in the need to analyze multifaceted inputs, including both unstructured text data (such as credit histories, financial reports, and market sentiment) and structured numerical data (like credit scores and income levels) (Lessmann et al., 2015). In this landscape, models like Bidirectional Encoder Representations from Transformers (BERT), Robustly Optimized BERT Pretraining Approach (RoBERTa), and Large Language Model Meta AI (LLaMA) 3.2 stand out for their ability to extract actionable insights from vast amounts of unstructured text, enabling more informed decision-making (Devlin et al., 2018; Liu et al., 2019; Touvron et al., 2023).

BERT, an acronym for Bidirectional Encoder Representations from Transformers, leverages bidirectional context through the attention mechanism inherent to the Transformer architecture. Its pretraining objectives, which encompass Masked Language Modeling and Next Sentence Prediction, are crucial for capturing nuanced linguistic patterns essential for accurate credit risk assessment (Devlin et al., 2018). By analyzing relationships within text, BERT can identify subtle shifts in language that may indicate potential credit risks.

Building on BERT's successes, Robustly Optimized BERT Pretraining Approach (RoBERTa) optimizes the training methodology by omitting certain tasks, such as Next Sentence Prediction, and incorporating dynamic masking strategies. These enhancements boost predictive accuracy and strengthen the model's ability to handle complex and large-scale datasets, thereby providing a more robust solution for credit risk classification (Liu et al., 2019). Large Language Model Meta AI (LLaMA) 3.2 is designed as a specialized model for deployment in low-resource environments. With a focus on efficiency, it employs optimized data selection and advanced tokenization techniques. Optimized data selection refers to the process of training the model on high-quality and relevant subsets of data rather than excessively large and noisy datasets, which improves learning efficiency and reduces computational cost. Advanced tokenization techniques, such as subword tokenization, split text into smaller meaningful units that allow the model to better handle rare or unseen words. For example, a word such as 'unpredictability' may be tokenized into sub-components like 'un', 'predict', and 'ability', enabling the model to understand its structure and meaning even if the full word was not frequently observed during training. These techniques contribute to improved performance while maintaining computational efficiency. This capability is particularly important in credit risk analysis, where efficiently processing and interpreting large volumes of financial data is crucial (Touvron et al., 2023).

Recent literature highlights that important credit-related information is often contained in unstructured text sources, such as executive comments, analyst reports, and financial news, which are not readily captured in structured datasets. Transformer-based large language models have been shown to address these limitations by improving the extraction and utilization of information from such unstructured data (Golec and Alabduljalil, 2026). Central to these transformer-based models is the attention mechanism, which enables them to assess the importance of various words within a given context effectively. While this feature significantly enhances performance, it also presents challenges in terms of memory resource demands, as these models can be computationally intensive. In conclusion, we will examine potential strategies and solutions aimed at alleviating these memory constraints, facilitating the effective use of these state-of-the-art NLP models in credit risk classification tasks.

### 2.1 Credit risk models

Credit risk models are used to estimate the potential financial loss that a credit institution, such as a bank or peer-to-peer lender, might incur if a borrower defaults on repaying a loan. The most important component of a credit risk model is the probability of default, which is usually estimated statistically by employing credit scoring models. (Pathak et al. 2023) examines how banks can adapt to manage credit risk amid economic uncertainty caused by rising interest rates, inflation, and geopolitical tensions. (Heng and Subramanian 2022) provides an in depth examination of how machine learning enhances credit risk assessment and the role of Explainable AI in addressing its limitations. It highlights the high performance of machine learning algorithms in predicting credit risk, while emphasizing their lack of interpretability. The review discusses Explainable AI techniques like LIME and SHAP, which improve transparency by explaining model decisions, thereby fostering trust and ensuring fairness in lending practices.

(Jeyakarthic and Ramesh 2023) introduces the GPDBN-CRA model as a tool to assist financial institutions in evaluating loan applications. This innovative model integrates a Dynamic Bayesian Network to classify credit risk based on customer data, along with Genetic Programming for hyperparameter tuning, which enhances overall performance. Genetic programming is then used to optimize the parameters, leading to improved prediction accuracy. (Hassija et al. 2020) proposes a novel approach to credit scoring by integrating blockchain technology and prospect theory. Blockchain ensures a secure, transparent, and tamper-proof credit scoring system, reducing reliance on centralized credit bureaus and enhancing data security. This innovative approach offers a secure, efficient method for assessing credit risk, potentially improving lending outcomes and minimizing defaults. (Jukna 2022) examines different credit scoring models to help financial institutions select the best method. It compares traditional techniques like logistic regression with advanced machine learning models, evaluating their accuracy, interpretability, and efficiency. The study highlights the practical implications of these models for various credit risk scenarios and explores trends like big data and explainable AI to improve transparency and trust in credit scoring.

### 2.2 Natural language processing

Natural Language Processing is centered on enabling computers to understand and process human languages. These models are crafted to learn a language's structure, syntax, and linguistic patterns or specific tasks by training on extensive text datasets relevant to the target language and objective (Brown et al., 2020). The training of such models typically consists of three main steps. First, the text is segmented into smaller, manageable units through a process known as tokenization. These tokens are then transformed into a numerical format referred to as word embeddings, allowing for a more nuanced representation that supports mathematical operations (Devlin et al., 2018). Finally, a language model is trained using this tokenized and embedded dataset (Brown et al., 2020).

#### 2.2.1 Language modeling

A language model is a statistical representation of the probabilities associated with sequences of words or tokens. Based on a corpus of known words, it assigns likelihoods to each possible subsequent sequence of words or tokens (*t*_1_, *t*_2_, …, *t*_*n*_) in a particular language *L*. As shown in Equation 1, language models estimate the joint probability of a token sequence by decomposing it into a product of conditional probabilities. In essence, it acts as a predictive model that identifies the most probable next words in a given context (Brown et al., 2020).


$$\begin{array}{l} P\left(t_{1},t_{2},\ldots ,t_{n}\right)=p\left(t_{1}\right)p\left(t_{2}|t_{1}\right)\cdots p\left(t_{n}|t_{1},t_{2},\ldots ,t_{n-1}\right)\\ =\overset{n}{\underset{i=1}{\prod }}p\left(t_{i}|t_{1},t_{2},\ldots ,t_{i-1}\right) \end{array}$$ (1)

Training a language model involves utilizing a large unlabeled dataset to establish a foundational understanding of language. Subsequently, these models can be fine-tuned for specific downstream tasks using labeled data. The classical language modeling objective focuses on learning a left-to-right context, predicting the conditional probability of all preceding tokens *t*_1_, *t*_2_, …, *t*_*i*−1_ for a given token *t*_*i*_. This traditional approach, which only generates predictions based on previously encountered words, is known as auto-regressive or unidirectional. It is particularly well-suited for tasks related to language generation (Brown et al., 2020).

A prevalent learning objective in NLP is the masked language model (MLM) objective. In this method, certain words or tokens within a sentence are randomly masked, and the model's goal is to predict these omitted tokens. By examining the context surrounding the masked words, the model assigns probabilities to the most likely candidates for replacement. This language modeling technique enables the model to learn in both left-to-right and right-to-left directions, facilitating what is known as bidirectional reasoning. This capability is particularly advantageous for sentence-level tasks, including text classification, named entity recognition, sentence analysis, and question answering (Devlin et al., 2018).

#### 2.2.2 Encoder-decoder architectures

The Encoder-Decoder architecture is a neural network design for sequence modeling, particularly in NLP tasks like sentiment analysis and text classification, where input lengths can vary while outputs remain fixed. This architecture transforms input sequences of arbitrary length into output sequences (Pakdaman et al., 2025). Traditional models struggle with this variability, requiring fixed lengths for training. The Encoder-Decoder consists of two networks, an encoder that converts an input sequence into a fixed-length vector representation, and a decoder that generates the output sequence from this vector. Both networks are trained together to accurately encode and reconstruct the target sequence.

### 2.3 The transformer model

Several pre-trained Transformer-based models, including LLaMA 3.2 (Touvron et al., 2023), RoBERTa (Liu et al., 2019), and BERT (Devlin et al., 2018), have set new standards for performance in various NLP applications since their introduction. RoBERTa enhances BERT's architecture by employing improved training methods, while LLaMA 3.2 focuses on efficiency and scalability in low-resource settings. The success of these models is largely attributed to their effective use of the attention mechanism, which enables strong and context-aware language understanding.

#### 2.3.1 BERT

**Figure 1:**
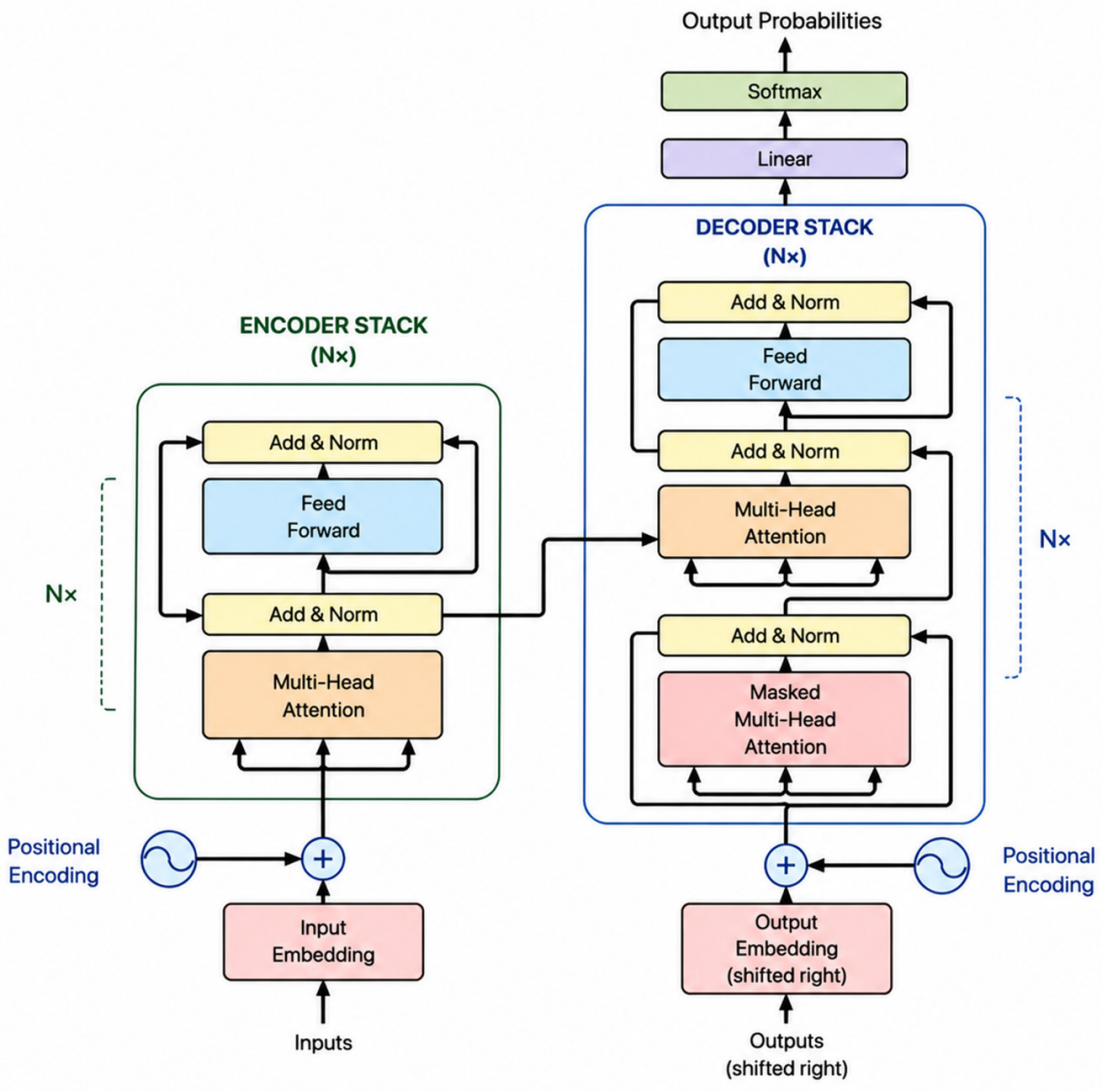
Figure depicting the BERT model architecture. The figure is adapted from (Rustam et al. 2024), who developed a classification method for lecturer expertise areas using BERT.

Bidirectional Encoder Representations from Transformers (BERT) is a language representation model created by researchers at Google AI Language in 2018. BERT was developed using deep learning techniques in conjunction with various other approaches. The architecture illustrated in Figure 1 of the BERT model features a multi-layer bidirectional Transformer, which mirrors the original implementation of the Transformer but exclusively employs the encoder component (Rustam et al., 2024).

(Koroteev 2021) highlights that BERT has had a transformative effect on NLP, demonstrating strong performance across tasks such as text classification, sentiment analysis, named entity recognition, and question answering. This improvement is primarily attributed to BERT's bidirectional training approach, which allows the model to consider both left and right context simultaneously, unlike earlier models such as ELMo and GPT that process text in a unidirectional manner. As a result, BERT is able to capture deeper contextual relationships within text, leading to improved accuracy and generalization across a wide range of NLP tasks. This contextual understanding enables the model to better interpret ambiguous words and complex sentence structures, which contributes to its superior performance compared to earlier architectures. The study by (Mann et al. 2023) explores the use of an Enhanced BERT model to enhance sentiment analysis of Twitter data. Utilizing the Kaggle SMILE dataset, the research tailors BERT to accommodate the informal nature of tweets, which frequently incorporate slang, acronyms, and emoticons. The Enhanced BERT model achieved an impressive accuracy rate of 96% in discerning sentiments such as happiness and sadness, highlighting its effectiveness. This research emphasizes the potential of Enhanced BERT in precisely analyzing the sentiments expressed in short, and informal texts.

Highlights that BERT has had a transformative effect on NLP, demonstrating strong performance across tasks such as text classification, sentiment analysis, named entity recognition, and question answering. This improvement is primarily attributed to BERT's bidirectional training approach, which allows the model to consider both left and right context simultaneously, unlike earlier models such as ELMo and GPT that process text in a unidirectional manner. As a result, BERT is able to capture deeper contextual relationships within text, leading to improved accuracy and generalization across a wide range of NLP tasks. This contextual understanding enables the model to better interpret ambiguous words and complex sentence structures, which contributes to its superior performance compared to earlier architectures.

#### 2.3.2 RoBERTa

**Figure 2:**
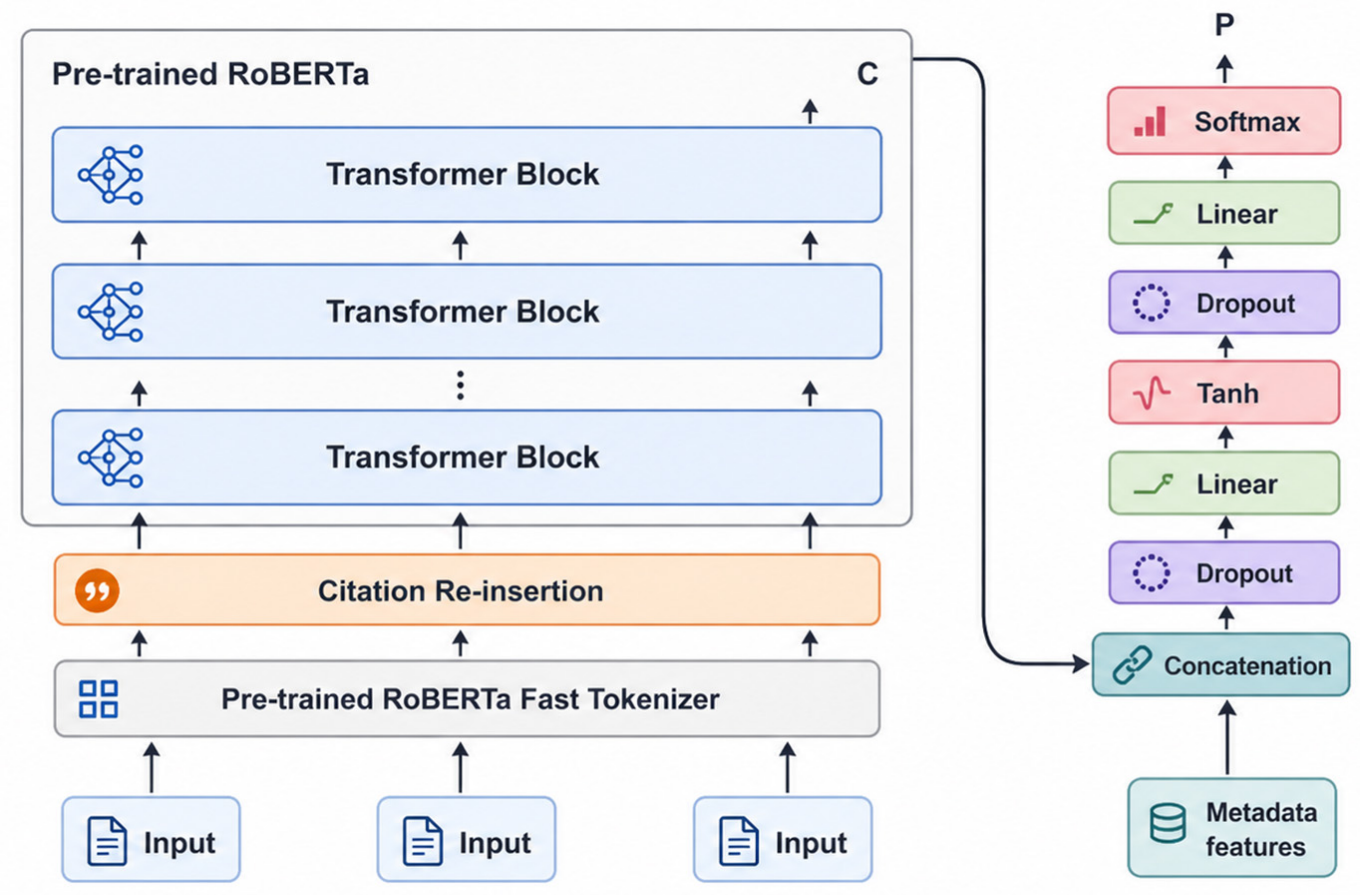
Figure depicting the RoBERTa model architecture. The figure is adapted from (Kumari 2023), who described modifications made to BERT for NLP in the blog post RoBERTa: A Modified BERT Model for NLP.

RoBERTa is an advanced NLP model created by Facebook AI in 2019, based on the BERT framework with several improvements aimed at enhancing its effectiveness (Chen et al., 2023). The architecture of the RoBERTa model, as modified from (Kumari 2023), is depicted in Figure 2.

#### 2.3.3 LLAMA

**Figure 3:**
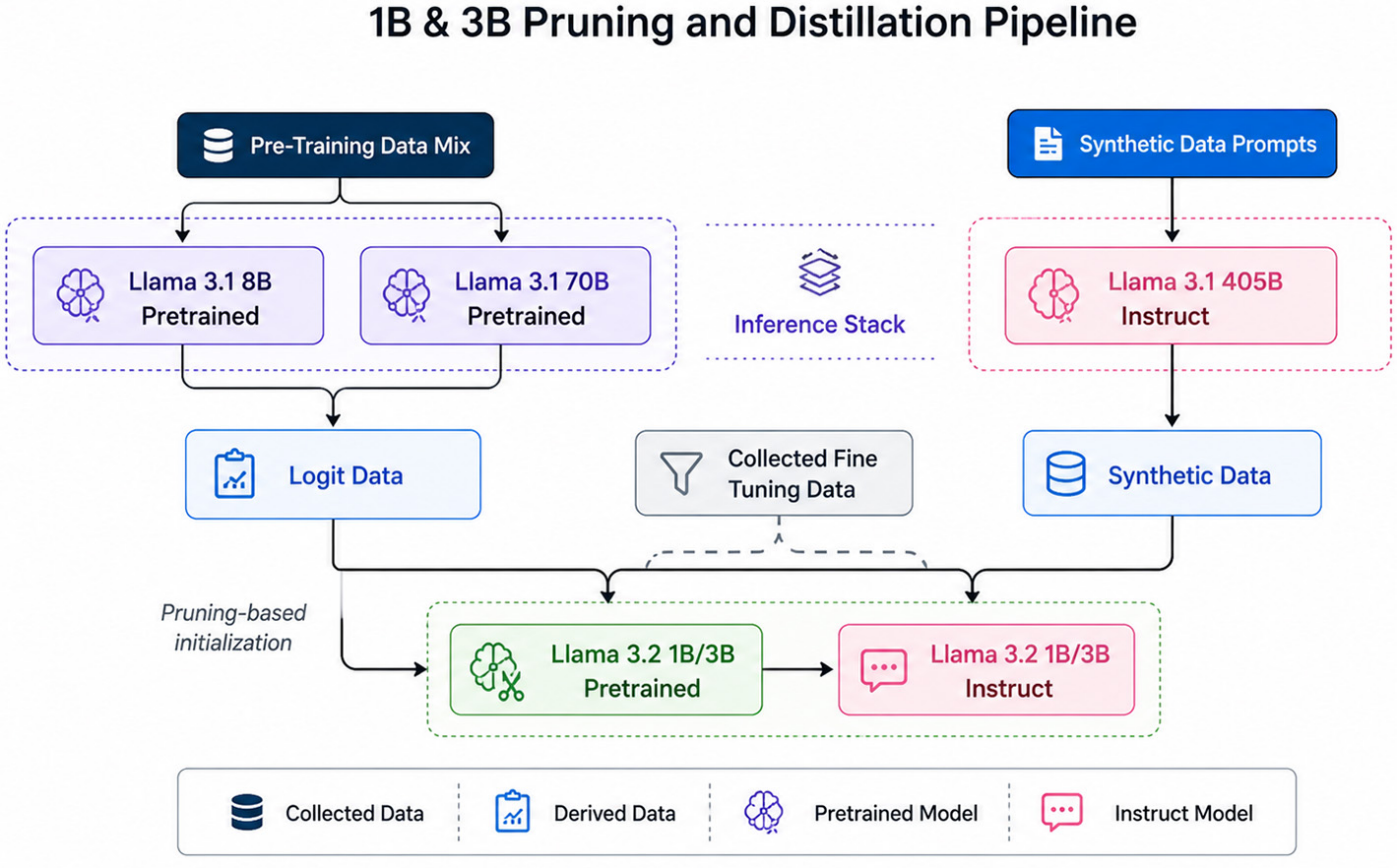
Figure depicting the LLaMA 3.2 model architecture. The figure is adapted from the blog post, From Vision to Edge: Meta's LLaMA 3.2 explained (Encord 2024), which provides an overview of its advancements and applications.

The LLaMA 3.2 − 1*B* model, illustrated in Figure 3, is a key component of the Large Language Model Meta AI (LLaMA) series, functioning as a robust tool for a wide range of NLP tasks. With 1 billion parameters, it effectively balances computational efficiency and performance, making it well-suited for both research and practical applications, especially in resource-constrained environments. Optimized for tasks such as text generation, summarization, and question answering, LLaMA 3.2 − 1*B* delivers impressive results while maintaining a lightweight profile compared to larger models in the series. Its design is tailored for users seeking an effective model that does not demand extensive computational power (Hugging Face, 2024).

(Stefan and Brad 2024) highlights the exceptional performance of LLaMA 3 in identifying subtle misinformation. It outperformed state-of-the-art competitors, achieving superior accuracy and precision because of its advanced contextual understanding and adaptability to complex narratives. Additionally, LLaMA 3 demonstrated remarkable efficiency in processing large datasets, rendering it suitable for real-time applications. Notably, the model demonstrated reasoning capabilities similar to human judgment, significantly reducing both false positives and false negatives. These findings emphasize its potential as a powerful tool for combating misinformation. (Sarfati et al. 2024) discusses how large language models (LLMs) emulate human cognitive processes like reasoning and decision-making. It highlights advancements such as multi-layer attention mechanisms and transformer networks that enable effective handling of complex language tasks. The paper also addresses the importance of improving model interpretability and considers ethical issues related to bias, fairness, and misuse, while emphasizing LLMs' transformative potential in sectors like healthcare, finance, and education.

## 3 Comparative analysis of different models and datasets

Table 2 outlines the datasets employed in the reviewed studies. These datasets span from unstructured sources like financial tweets and news articles to structured stock market time series and legal judgments. Most are annotated with sentiment or emotion labels, while others use inferred labels like risk tolerance or psychometric traits. A few works rely on synthetic or proprietary data, limiting reproducibility and cross-study comparability. The table categorizes sources by type, language, size (where available), and annotation strategy, providing insight into the diversity and challenges of data collection in financial NLP. It also clarifies each dataset's intended analytical purpose, such as credit scoring, supplier evaluation, or emotion classification.

**Table 2:** Dataset overview from reviewed articles on NLP for credit and financial risk.

Source	Data type	Language	Annotation	Purpose
P2P lending descriptions (Yuan and Wei, 2024)	Unstructured loan text	English	No manual annotation; LLM-derived risk indicators	Use borrower-written descriptions to construct interpretable credit risk indicators via Large Language Models (LLMs)
Social media posts (Yang et al., 2022)	User-generated text from P2P lending platforms	English	Self-supervised psychometric labeling	Infer borrowers' psychological traits to support credit risk evaluation
P2P loan applications (Brahma et al., 2021)	User-generated loan descriptions	English	Labeled with default outcomes	Predict credit default using deep learning on textual applications
Bank financial data (Tao et al., 2025)	Structured financial indicators	Not specified	Labeled with default outcomes	Predict financial risk using structured data and deep learning models
Financial news articles (Adhikari et al., 2023)	Financial news headlines	English	Labeled with sentiment polarity	Improve sentiment analysis accuracy using hybrid word embeddings and explainable AI techniques
Financial text data (Lin and Liao, 2024)	Financial documents	Not specified	Annotated with sentiment labels using lexicon-based prompts	Enhance financial sentiment analysis through lexicon-based prompt methods
Financial twitter data (García-Méndez et al., 2023)	Tweets mentioning financial assets	English	Annotated with ‘opportunity' and ‘precaution' emotions per asset	Detect asset-specific financial emotions for market screening and decision-making
Financial reports and market data (Che et al., 2024)	Annual reports, financial indicators, and market data	English	Labeled with financial distress outcomes	Predict financial distress using an attentive and interpretable multimodal framework
Financial transaction graphs (Boyapati and Aygun, 2025)	Transactional data represented as graphs	English	Labeled with fraudulent and non-fraudulent classes	Detect fraud in imbalanced datasets using graph neural networks

Table 3 presents a comparative overview of NLP models applied in financial and credit risk analysis. The models range from transformer-based architectures to convolutional and hybrid deep learning structures, each tailored to a specific problem such as sentiment analysis, credit scoring, or entity disambiguation. Most architectures integrate attention mechanisms or hybrid word embeddings to address polysemy, interpretability, and contextual representation in financial text. Some models, such as PsyCredit and PLWL, incorporate explainability techniques like LRP or dictionary filtering to enhance transparency. Performance across models varies, often based on task and dataset, but many demonstrate significant improvement over traditional baselines. The table also highlights which models emphasize explainability, accuracy, or application novelty.

**Table 3:** Model overview from reviewed articles on NLP for credit and financial risk.

Model	Architecture	Data source	Input features	Performance
LLM risk indicator (Yuan and Wei, 2024)	Zero-shot LLM (GPT-3.5/BERT baseline)	P2P loan descriptions	Loan purpose and intention text authored by borrowers	Risk scores correlated with actual defaults; outperforms lexical baselines
PsyCredit (Yang et al., 2022)	Deep learning with LRP (Layer-wise Relevance Propagation)	Social media posts from P2P platforms	Linguistic psychometric signals	Outperformed baseline credit risk scoring models
Credit default predictor (Brahma et al., 2021)	Deep Neural Network (DNN)	P2P loan applications	Descriptive features from borrower text	Achieved better accuracy than traditional financial models
LocalGov debt risk model (Guo et al., 2022)	Deep learning NLP pipeline with sentiment analysis	Government work reports	Sentiment-based risk indicators	Effectively identified high-risk geographic regions
grcForest_XGB (Tao et al., 2025)	Ensemble of grcForest and XGBoost	Bank financial datasets	Structured financial indicators	Delivered superior predictive performance among benchmarked models
MobilePay risk KG (Xia et al., 2022)	Deep learning for unsupervised knowledge graph construction	Mobile payment policy documents	Extracted entities and relationship triples from text	Enabled visual risk analysis and policy linkage via knowledge graphs
Hybrid word representation (Adhikari et al., 2023)	CNN with attention using hybrid word embeddings	Financial news headlines	Embeddings capturing semantic, syntactic, and polysemous cues	Achieved stronger sentiment classification than baseline approaches
CNN Risk Profiler (Xing, 2024)	CNN + psycholinguistic signals	Synthetic user-generated text	Linguistic markers + embeddings	Micro-F1 approx 0.51
BERTeley (Chagnon et al., 2024)	Transformer-based topic modeling with pre-trained scientific language models	Scientific article corpora	Sentence embeddings from scientific texts	Demonstrated superior coherence and topic quality
LLM4Jobs (Li et al., 2025)	Unsupervised LLM-based framework	Job postings and resumes	Raw textual data	Outperformed existing unsupervised methods

Our comparative synthesis highlights how transformer-based models especially BERT and RoBERTa demonstrate superior performance across tasks such as sentiment classification and credit scoring. Hybrid approaches combining CNNs or GRUs with transformers further improve accuracy and adaptability, especially in imbalanced datasets. While LLaMA models show promise in low-resource contexts, BERT remains the most widely adopted. Notably, explainability is still lacking in many high performing models, with limited deployment of interpretability tools like SHAP or LIME in financial applications (Golec and Alabduljalil, 2026).

## 4 Methodology

This study uses a Systematic Literature Review (SLR) methodology to systematically identify, evaluate, and synthesize academic research on the integration of NLP models, specifically transformer-based architectures such as BERT, RoBERTa, and LLaMA, in credit risk classification tasks. Studies from adjacent domains were retained where they demonstrated transferable transformer-based methodologies relevant to credit risk modeling. The SLR uses Python based tools to ensure methodological transparency and reproducibility by integrating structured database querying with automated processes for filtering, cleaning, and scoring semantic similarity. To ensure comprehensive coverage, scholarly articles were sourced from three prominent databases: Scopus, ScienceDirect, and Web of Science. A standardized Boolean search string was crafted to explore these sources, focusing on papers on credit risk, financial risk, or loan default classification utilizing NLP or text mining techniques. The search was confined to English language publications released between 2015 and 2025. The query structure for Scopus is as follows 10:

TITLE-ABS-KEY (
    (credit risk
     OR financial risk
     OR loan default
     OR credit scoring)
    AND
    (natural language processing
     OR NLP
     OR text mining)
    AND
    (classification
     OR prediction)
)
AND PUBYEAR > 2015
AND PUBYEAR < 2025
AND (
    EXCLUDE(DOCTYPE, cp)
    OR EXCLUDE(DOCTYPE, re)
    OR EXCLUDE(DOCTYPE, cr)
    OR EXCLUDE(DOCTYPE, sh)
    OR EXCLUDE(DOCTYPE, ed)
)
AND (
    EXCLUDE(LANGUAGE, Chinese)
)

ScienceDirect's query was formulated using the basic search bar and fine-tuned for optimal keyword matching. Although the interface lacks the Boolean complexity found in platforms like Scopus, it allows for a direct logical structure that effectively yields relevant peer-reviewed results. The search targeted the comprehensive full-text repository of Elsevier publications to locate articles that discuss NLP in the context of credit risk. The logical structure emphasized classification and prediction, specifically incorporating credit-related terminology. The search was also limited to publications in the English language that were released between 2015 and 2025. The following query demonstrates the syntax compatible with ScienceDirect's search functionality 11:

(
    credit risk
    OR financial risk
    OR loan default
    OR credit scoring
)
AND (
    natural language processing
    OR NLP
    OR text mining
)
AND (
    classification
    OR prediction
)
AND publicationYear:[2015 TO 2025]
AND language:English

The Web of Science platform offers a Topic Search (TS) feature for thorough querying of article titles, abstracts, author keywords, and Keywords Plus. The final query structure has been refined for universal compatibility and is implemented through the core collection interface. To narrow down the results, only publications from 2015 to 2025 in English were considered, and document types such as reviews and editorial materials were excluded. Below is the corresponding query that adheres to Web of Science's default logic syntax 12:

TS = (
    credit risk
    OR financial risk
    OR loan default
    OR credit scoring
)
AND TS = (
    natural language processing
    OR NLP
    OR text mining
)
AND TS = (
    classification
    OR prediction
)
AND PY = (2015-2025)
AND LA = (English)
NOT DT = (
    Meeting Abstract
    OR Editorial Material
    OR Review
)

### 4.1 Inclusion and exclusion criteria

To ensure thematic and methodological consistency, explicit eligibility criteria were applied, as summarized in Table 4.

**Table 4:** Inclusion and exclusion criteria for study selection.

Criterion	Inclusion	Exclusion
Research focus	Studies applying transformer-based NLP models (e.g., BERT, RoBERTa, LLaMA) to credit risk or financial risk prediction	Studies unrelated to credit risk, financial risk, or not involving NLP/textual analysis
Model type	Transformer-based or hybrid NLP models	Traditional models without NLP components
Publication period	Published between 2015 and 2025	Published before 2015
Publication type	Peer-reviewed journal articles and conference papers	Editorials, review papers, position papers, or non-empirical studies
Language	English-language publications	Non-English publications
Data modality	Use of unstructured textual data (alone or combined with structured data)	Studies relying exclusively on structured numerical data

### 4.2 Screening, removal of duplication, and semantic filtering

**Figure 4:**
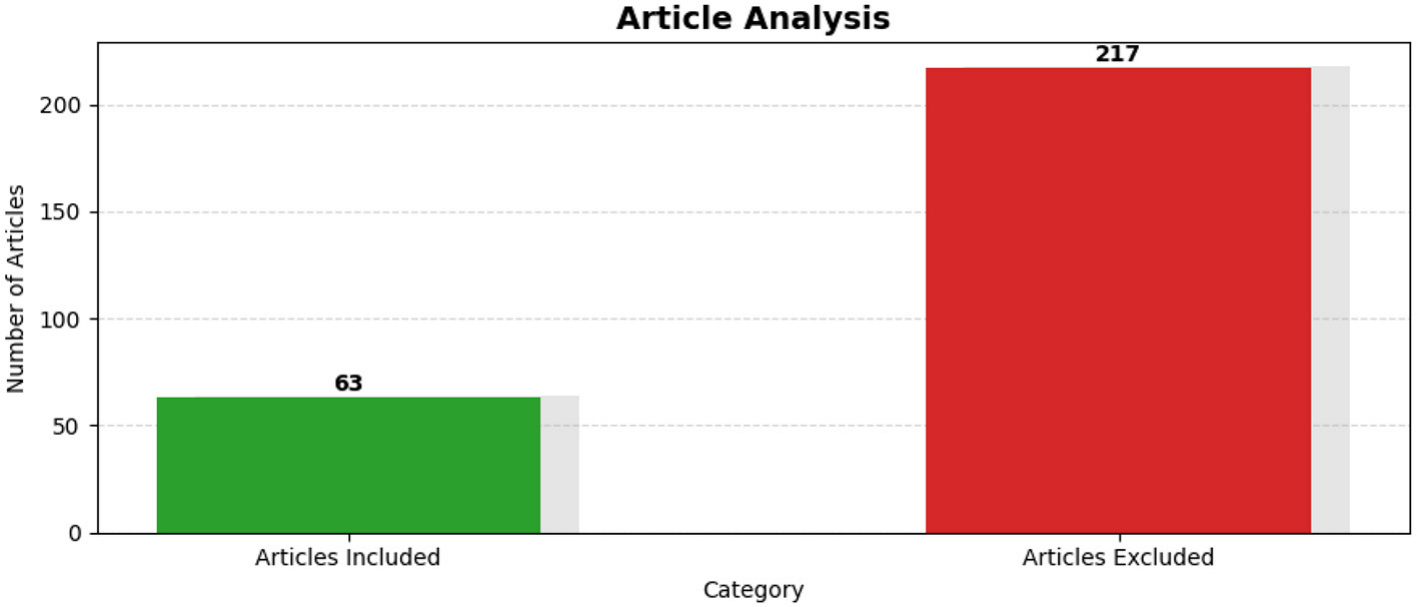
Bar chart comparing the number of included vs. excluded articles after semantic filtering.

The initial search produced 284 unique records across the databases, which were then exported in BibTeX format and consolidated into a single bibliography. Following this export, a semantic filtering process was implemented to evaluate the relevance of each article against a predefined research theme. Of the total records, 217 articles were excluded due to low semantic similarity, while 63 articles met the inclusion criteria. Figure 4 visualizes the proportions of included versus excluded articles, providing a clear representation of the data refinement process that underlies the methodological rigor of the review.

A Python script utilizing the bibtexparserlibrary was used to load and parse the entries, while duplicates were identified and eliminated by string normalization of titles and unique identifiers.

import bibtexparser
from bibtexparser.bparser
import BibTexParser
from bibtexparser.customization
import homogenize_latex_encoding

def normalize(text):
    return text.strip().lower().
    replace
    ('\n', '')
    if text else ''

def remove_duplicates(entries):
    seen_titles = set()
    unique_entries = []
    for entry in entries:
        title = normalize(entry.get
        ('title', ''))
        if title not in seen_titles:
            seen_titles.add(title)
            unique_entries.append
            (entry)
    return unique_entries

After removal of duplications, 280 unique records remained. To refine the corpus to topically relevant studies, a semantic filtering step was introduced. Using the SentenceTransformer model all-MiniLM-L6-v2, Cosine similarity was calculated between the embeddings of each article derived from its title and keywords, and a target embedding that represents the central theme of the review. To reduce noise and computational costs during the initial filtering, abstracts despite containing important context were excluded. Future refinements may consider a tri-modal approach that includes abstracts for enhanced coverage. The threshold of 0.35 was established on the basis of preliminary experiments to ensure the inclusion of thematically relevant but semantically diverse articles. A sensitivity analysis of similarity scores (0.35–0.573) showed that lower thresholds (e.g., 0.30) increased the number of included studies but introduced partially related literature, while higher thresholds (e.g., 0.40) reduced the corpus size by excluding relevant but semantically diverse studies. For instance, highly ranked articles such as “PsyCredit: An interpretable deep learning-based credit assessment approach” (similarity = 0.573)

from sentence_transformers
import SentenceTransformer, util
import torch

model = SentenceTransformer
('all-MiniLM-L6-v2')

target_topic = (
 Optimising credit risk model
 classification
  using NLP,
    BERT, RoBERTa, and LLaMA
)
target_embedding = model.encode(
    target_topic, convert_to_tensor
    =True
)

**Figure 5:**
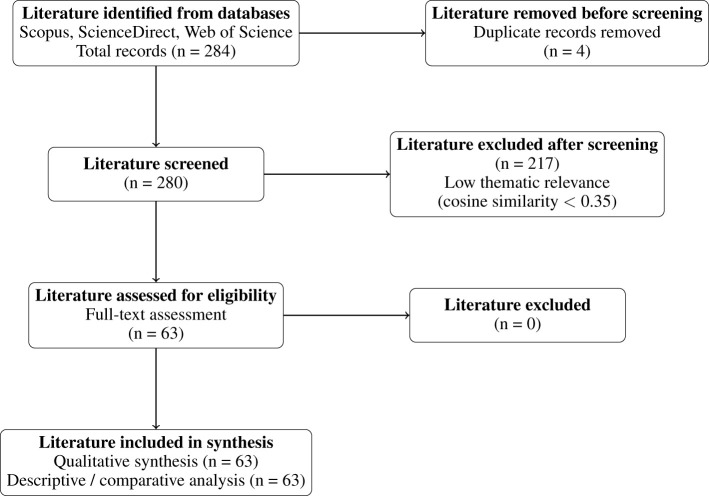
PRISMA 2020 flow diagram illustrating the identification, screening, eligibility assessment, and inclusion of studies in the systematic review of transformer-based NLP models for credit risk prediction (Page et al., 2021). The semantic similarity filtering procedure consolidates the traditional screening and eligibility stages into a single automated step. As a result, no additional exclusions were done during the full-text eligibility phase.

SIMILARITY_THRESHOLD = 0.35
similar_articles = []

for entry in unique_entries:
    combined_text = "".join([
        entry.get(title, ""),
        entry.get(keywords, "")
    ])
    if not combined_text.strip():
        continue

    entry_embedding = model.encode(
        combined_text, convert_to_
        tensor=True
    )
    similarity = util.cos_sim(
        target_embedding, entry_
        embedding
    ).item()

    if similarity >= SIMILARITY_
    THRESHOLD:
        entry["similarity"] = round
        (similarity, 3)
        similar_articles.append(entry)

and “Automated mortgage origination delay detection from textual conversations” (similarity = 0.565) demonstrate strong thematic alignment, while studies closer to the lower bound, such as “BalancerGNN: Graph Neural Networks for imbalanced datasets: A case study on fraud detection” (similarity = 0.35), capture relevant but more methodologically diverse approaches within the broader financial risk domain. Consequently, only articles that met or surpassed a semantic similarity score of 0.35 were selected for detailed analysis, resulting in a curated collection of 63 studies. This rigorous filtering process ensured that the literature reviewed closely aligned with the thematic focus on credit risk modeling using NLP while maintaining methodological diversity. In the interest of methodological transparency, the screening process adhered to PRISMA principles. The key phases encompassed article identification, removal of duplicates, semantic evaluation, and final inclusion based on relevance. Each stage contributed to refining the dataset into a robust evidence base. The distribution and flow of articles through these phases are visually represented in the Sankey-style PRISMA diagram provided in Figure 5.

**Figure 6:**
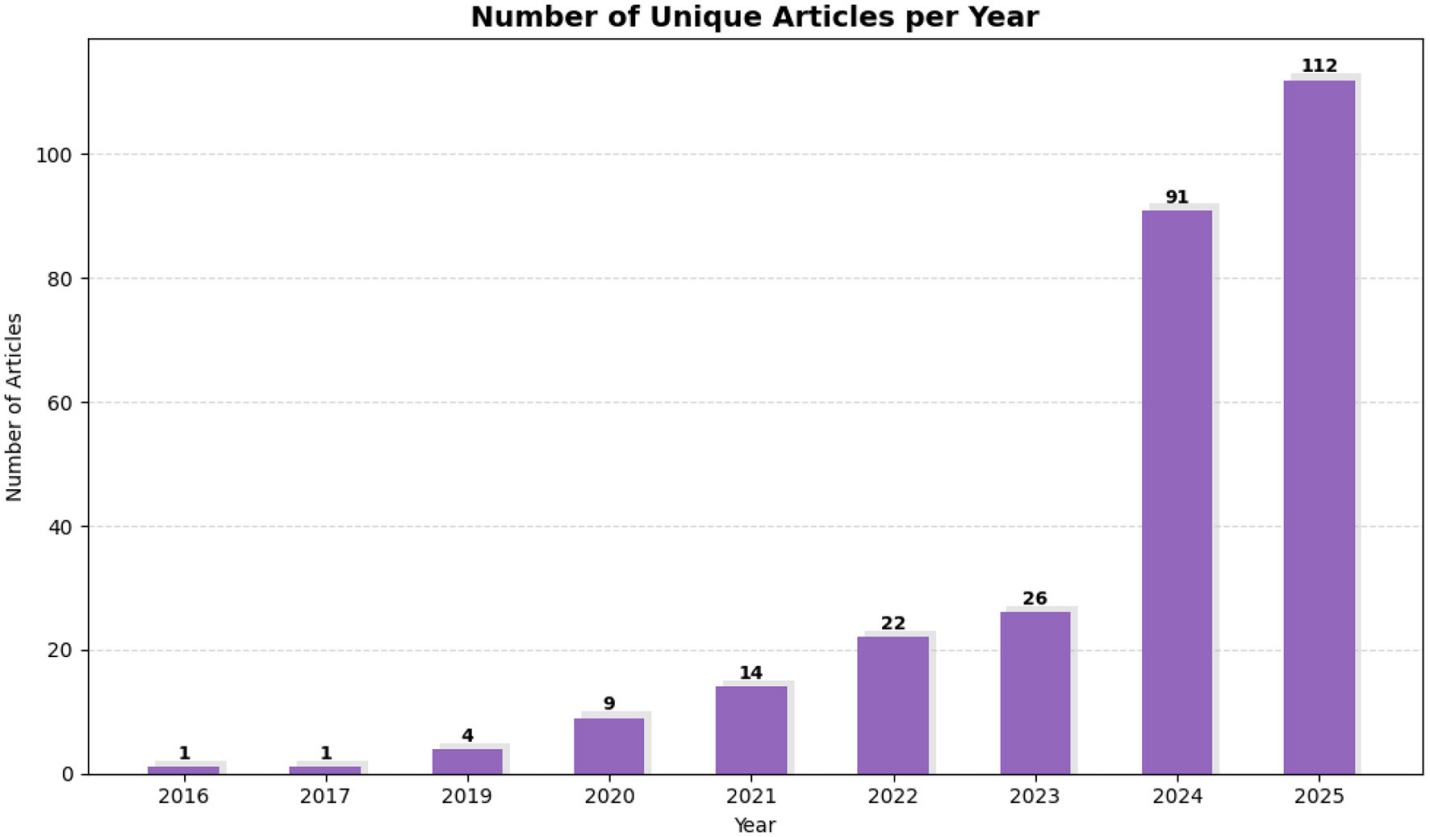
Distribution and publication trends of selected studies over the years, illustrating the increasing academic interest in credit risk classification using NLP techniques.

Figure 6, which illustrates publication trends, reveals a clear upward trajectory in relevant research output, particularly beginning in 2020. This trend highlights an increasing scholarly emphasis on the application of NLP within financial risk modeling. As depicted in Figure 6, the peak anticipated in 2025 signals a significant recent surge in academic engagement, likely driven by advancements in transformer-based models and the growing accessibility of financial text data. This pattern also reflects the broader integration of machine learning in finance, coinciding with various regulatory and technological changes. Overall, the temporal analysis underscores the relevance and importance of this study within the dynamic research landscape.

**Figure 7:**
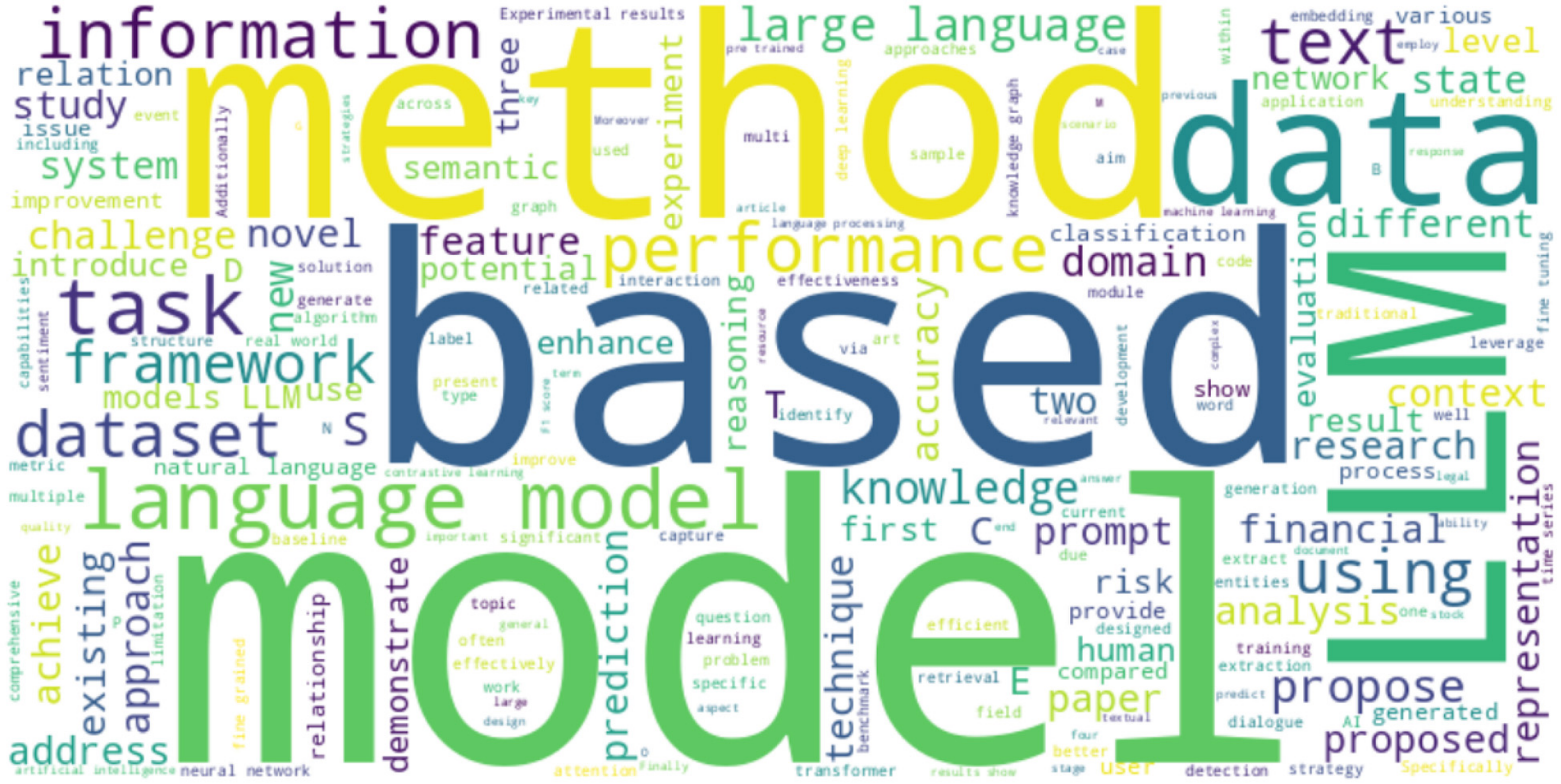
Word cloud illustrating the most frequently occurring terms in the titles, abstracts, and keywords of the screened literature, highlighting key themes such as LLM, methods, models, classification, prediction, and risk.

The thematic structure of the reviewed literature is visually summarized in a word cloud (Figure 7), which was generated by aggregating text from article titles and keywords. This visualization technique enabled the identification of key thematic patterns within the filtered corpus. Notable terms such as “LLM,” “classification,” “risk,” “based,” “language model,” and “prediction” appeared with high frequency, underscoring the central focus areas of the included studies. The prominence of these keywords highlights the importance of large language models and predictive analytics in the context of credit risk modeling. This thematic consistency further validates the article selection process and reinforces the alignment of the literature with the study's research objectives.

The integration of programmatic processing with scholarly review processes enhances methodological rigor and transparency. This hybrid approach utilizing NLP models, semantic embeddings, and structured screening frameworks ensures the inclusion of only the most relevant and high-quality studies. The resulting corpus serves as a solid foundation for analyzing current trends, assessing model efficacy, and understanding the evolving landscape of NLP in credit risk modeling.

### 4.3 Quality assessment

Each of the 63 included studies was systematically evaluated for methodological quality and potential risk of bias using an adapted GRADE-based assessment framework. The evaluation focused on several core criteria to ensure rigor and comparability across studies. First, dataset characteristics were examined, including dataset size, representativeness, and whether the data were publicly accessible or proprietary. Second, we assessed the clarity and reproducibility of the reported methodologies, including transparency in model architecture descriptions, preprocessing steps, and training procedures. Third, studies were evaluated on whether they included appropriate baseline model comparisons, particularly against traditional statistical or non-transformer machine learning approaches. Finally, the completeness and transparency of reported evaluation metrics such as accuracy, AUC, F1-score, recall, precision, and other task-specific measures were examined to determine the robustness and validity of reported findings. This structured assessment ensured that conclusions drawn from the literature were grounded in methodologically sound and reproducible research.

### 4.4 Data extraction and synthesis

For each of the 63 eligible articles included in the final synthesis, a structured data extraction process was conducted to ensure consistency and comparability. Extracted information included dataset characteristics such as type (e.g., loan descriptions, financial news, social media data, transaction records), dataset size where available, and language of the textual data. Detailed information on model architectures and training strategies was recorded, including whether transformer-based, hybrid, multimodal, or ensemble approaches were used, as well as fine-tuning or zero-shot strategies where applicable. Evaluation metrics and reported performance outcomes were systematically documented to facilitate cross-study comparison. Additionally, we extracted information regarding the use of explainability, fairness, or bias-mitigation techniques, including methods such as SHAP, LIME, LRP, or interpretable modeling frameworks. Finally, reported limitations, ethical considerations, and regulatory implications discussed by the authors were captured to provide a comprehensive synthesis of both technical performance and broader deployment concerns. This structured extraction approach enabled a coherent analytical comparison across diverse methodological designs.

## 5 Data analysis and discussion

### 5.1 Data analysis

(Shen and Wu 2025) TCN-DilateFormer model shows high precision, recall, F1 score, and specificity, outperforming classical and state-of-the-art models in credit risk analysis. Parameter optimization revealed [1,2,4,8] as the optimal dilation factor combination and the original setting of [1,2,4] as the best scale configuration. The model exhibits tolerance to learning rate changes and low sensitivity to Gaussian noise variations, demonstrating robustness. Statistical tests confirm TCN-DilateFormer's superior performance compared to other models. (Barrak et al. 2022) utilized multiple data sources, including the Airudi dataset, web scraping, and the RecSys Challenge 2017 dataset. The Airudi dataset contains job descriptions and resumes, while web scraping provided real-world postings and candidate profiles. The authors used deep contextualized word embeddings and language models to focus on key feature extraction, such as skills and experience. A baseline model with transformer-based language models was developed to match candidates to job descriptions by assessing feature similarities. Performance was evaluated using metrics like Normalized Discounted Cumulative Gain (NDCG) and Mean Reciprocal Rank (MRR), improving accuracy and explainability. The system ensured fair and unbiased matching, with traceability features to monitor decision-making and address discrimination concerns.

The study (Rustam et al. 2024) introduced a novel method for classifying the areas of expertise of lecturers by analyzing the titles and abstracts of their scientific publications. By leveraging the Bidirectional Encoder Representations from Transformers BERT model, the researchers were able to convert unstructured text data into meaningful vector representations. This advanced deep learning model effectively classified the publications into 24 distinct scientific categories, achieving an impressive accuracy of 95.03% on the training data and 92.88% on the test data. This approach offers a more objective and data-driven framework for mapping lecturers' expertise, thereby enhancing the precision of academic talent management systems. (Roshanzamir et al. 2021) evaluated models using picture description test transcripts and the BERTLarge model with logistic regression achieved 88.08% classification accuracy. This improves the state-of-the-art by 2.48%. The sentence-level BERTLarge embedding method achieved the highest accuracy score 88.08%. The models based on the BERT family of embedders performed better than others. The best model has a Pearson correlation of 0.78 and a Spearman's rank correlation of 0.81 for the training phase. According to (Jin and Zhang 2024) the CNN-Transformer-WT model achieved a prediction accuracy of 0.969 on the Kaggle Dataset, 0.953, also surpassing other models. The risk recall for the CNN-Transformer-WT model was 0.947, higher than the GRU and BERT models. The model's comprehensive F1 score reached 0.955, exceeding other models' scores. The results confirm the effectiveness and superiority of the CNN-Transformer-WT model in financial risk prediction tasks.

The research (Wang et al. 2019) showed that LSTM-based models outperform traditional methods in credit risk assessment using P2P lending user data. The bidirectional LSTM (BLSTM) performs better than standard LSTM, as it captures sequence information from different directions. The attention-based LSTM (AM-LSTM) excels compared to standard BLSTM and BLSTM-Meanpool. The AM-LSTM model improved the KS value by 10.3% and AUC value by 3.08%. Models incorporating operation behavior data outperformed those without it, improving the KS value by 10.7% and the AUC value by 5.65%. This shows the advantages of using operation behavioral data in credit scoring. (Shen et al. 2025) suggests that NLP models can predict triage scores, need for admission, and critical illness with high accuracy, and can map free-text complaints to structured data. Multimodal models, incorporating both structured and free-text data, generally outperform models using only structured data. Free-text data alone can predict admission with high accuracy. Some studies show NLP models are more accurate than nurses in assigning triage scores. A deployed NLP model improved structured data capture. However, most studies have a high risk of bias, and few have been implemented in clinical practice.

In their study, (Mahbobi et al. 2021) developed an algorithm to improve credit risk assessment, focusing on imbalanced datasets where loan defaults are a concern. They utilized a dataset of 30, 000 instances and used resampling techniques like SMOTE, SVM SMOTE, Random Undersampling, and ALL-KNN to enhance predictive accuracy. Various machine learning classifiers were used, including Deep Neural Networks, Support Vector Machines, K-Nearest Neighbors, and Artificial Neural Networks. The SVM model with ALL-KNN sampling achieved an impressive accuracy of 98.6% and a cross-entropy loss of 0.028. While emphasizing predictive accuracy, interpretable models like KNN and ANN also offered insights into the decision-making process, addressing concerns about fairness and explainability in credit risk assessments. This approach demonstrates the effectiveness of combining deep learning with resampling techniques for credit risk classification in imbalanced scenarios.

The paper (Tan et al. 2023) introduced a novel approach that combines the strengths of the Transformer-based RoBERTa model and Gated Recurrent Units (GRU) to improve sentiment analysis. The RoBERTa component effectively captures contextual word representations through its attention mechanism, while the GRU component adeptly models long-range dependencies in the text. To address challenges posed by imbalanced datasets, the authors employed data augmentation techniques, specifically oversampling minority classes using word embeddings, thereby enhancing the model's robustness and accuracy. Evaluations carried out on three widely used sentiment analysis datasets -IMDb, Sentiment140, and Twitter US Airline Sentiment - demonstrated the effectiveness of the model, achieving accuracies of 94.63%, 89.59% and 91.52%, respectively. These results underscore the potential of the RoBERTa-GRU hybrid model in advancing sentiment analysis tasks. (Chen et al. 2023) used the ELSTM-VC model, which combines an Extra Trees Classifier (ETC) with a Long Short-Term Memory (LSTM) network to detect explicit content in English song lyrics. They trained a dataset of 100 songs from Spotify, and the model achieved a 96% accuracy rate, outperforming existing machine learning models and encoding-decoding approaches. This automated method offers a more efficient and accurate solution for identifying inappropriate music content, protecting young listeners from exposure to explicit songs.

The authors of (Alrowais et al. 2024) developed a model that integrates the RoBERTa transformer with GloVe word embeddings to detect cyberbullying in tweets. This model was trained on a publicly available dataset focused on cyberbullying and achieved an impressive accuracy rate of 95%. It outperformed existing machine learning, deep learning, and transformer-based approaches that utilized FastText word embeddings. This research highlights the efficacy of combining transformer models with word embeddings to improve the detection of cyberbullying on social media platforms. The paper (Mann et al. 2023) investigated the application of an improved BERT model for sentiment analysis of tweets. They made use of the Kaggle SMILE dataset, which contains tweets labeled with various emotions, including happiness and sadness. Their enhanced BERT model achieved an impressive accuracy of 96% in classifying these sentiments, showcasing its effectiveness in comprehending the nuanced emotional content present in tweets.

(Roumeliotis et al. 2024) investigated how effective LLMs are at evaluating sentiment in product reviews from e-commerce sites. They assessed the performance of the GPT-3.5 and LLaMA-2 models, both in their pre-trained and fine-tuned versions, using a dataset of product reviews sourced from different e-commerce platforms. Their results showed that fine-tuning greatly improved the accuracy of the models in sentiment classification, with GPT-3.5 achieving an accuracy rate of 92% and LLaMA-2 obtaining 89%. This study highlights the potential for LLMs to automate sentiment analysis in e-commerce, which can help businesses gain a better understanding of customer feedback. The paper by (Jeyakarthic and Ramesh 2023) presented the GPDBN-CRA model, which combines genetic programming with dynamic Bayesian networks to improve the assessment of credit risk. This model aids financial institutions in making decisions on loan applications by standardizing customer data and effectively evaluating creditworthiness. The GPDBN-CRA model exhibited better performance than conventional credit risk assessment approaches, providing a more precise and efficient tool for financial decision-making.

According to (Song et al. 2024) the Monitoring and Early Warning Model Based on LSTM, Transformer, and Deep Learning model achieved a high accuracy across different datasets. 94.37% on the Fama-French Three-Factor Dataset and 93.84% on the CRSP Dataset. There are also significant improvements on the Compustat dataset with 95.13% accuracy and the World Bank dataset produced a 94.34% accuracy. The model's efficiency is evident through its low parameter count, short inference time of 213.58ms, and training time of 147.24 seconds on the Fama-French Three-Factor Dataset. Combining LSTM and Transformer boosts model accuracy and recall. The model can also detect risk signals earlier, enabling proactive risk management.

### 5.2 Discussion

The results of this systematic review indicate that the incorporation of NLP and Large Language Models (LLMs) into credit risk modeling signifies a noteworthy advancement in the way financial institutions analyze and interpret extensive amounts of unstructured data. Traditional models, which typically rely on structured inputs such as credit history, income level, and repayment behavior, are increasingly being enhanced and in some cases even surpassed by models that utilize textual sources like loan applications, social media posts, financial disclosures, and news articles (Wang et al., 2019; Jin and Zhang, 2024; Sanz-Guerrero and Arroyo, 2025; Yang et al., 2022; Adhikari et al., 2023; García-Méndez et al., 2023).

Among the most impactful models in natural language processing are BERT, RoBERTa, and LLaMA, which utilize attention mechanisms and pretraining strategies to draw out contextual and semantic insights from text. To consolidate these approaches, Table 5 presents an analytical synthesis of NLP and LLM-based models in credit risk modeling, highlighting their key contributions and limitations across model categories. These models excel in classification tasks related to credit scoring, default prediction, and fraud detection. For instance, BERT's bidirectional training facilitates a deeper comprehension of linguistic context, making it particularly effective at discerning nuanced sentiments within borrower narratives. RoBERTa enhances this capability with optimized pretraining and dynamic masking, while LLaMA provides scalability and efficiency suited for real-time risk assessment in resource-constrained environments (Touvron et al., 2023).

**Table 5:** Analytical synthesis of NLP and LLM-based approaches in credit risk modeling.

Model category	Representative models	Primary data sources	Key contributions	Key limitations
Transformer-based NLP	BERT, RoBERTa, LLaMA	Loan descriptions, financial news, social media, disclosures	High contextual understanding; improved default and sentiment prediction; strong performance across credit scoring tasks	Limited interpretability; high computational cost; weak regulatory alignment
Hybrid NLP models	RoBERTa-GRU, CNN-Transformer, BERT-LSTM	Textual data combined with time-series and structured financial features	Improved robustness; superior performance on imbalanced datasets; better temporal modeling	Increased architectural complexity; limited transparency in decision logic
Multimodal risk models	Text + numerical financial indicators	Financial statements, transaction histories, borrower narratives	Enhanced predictive accuracy; captures complementary risk signals across modalities	Data integration challenges; reproducibility issues due to proprietary datasets
Attention-based and graph models	ACWGAN-GPSA, RCMA, Graph Neural Networks	Legal documents, transactional graphs, policy texts	Reveals hidden risk patterns; effective fraud and supply chain risk detection	High training complexity; limited scalability; sparse explainability evaluation
Explainability-enhanced models	Models using LIME, SHAP, LRP	Structured and unstructured financial text	Improved transparency; partial regulatory compliance; enhanced trust	*Post-hoc* explanations may be unstable; limited faithfulness to model reasoning

In addition to these core models, the review highlights a trend toward hybrid architectures and multimodal frameworks that integrate NLP techniques with structured numerical features. Models like RoBERTa-GRU and CNN-Transformer hybrids demonstrate enhanced performance in sentiment analysis and risk classification, particularly when applied to intricate datasets that encompass both text and time-series data (Jin and Zhang, 2024). Attention-based models, such as ACWGAN-GPSA and the RCMA fraud detection system, exemplify how detailed modeling of financial documents and legal texts can reveal concealed risk signals (Wang et al., 2025; Boyapati and Aygun, 2025).

Despite these advancements, several challenges continue to exist. Ethical and regulatory considerations are vital for real world deployment of NLP-based credit risk models. Fairness in lending is a primary concern, particularly given evidence of algorithmic bias against certain demographic groups. Model interpretability remains a significant concern, particularly in regulated sectors such as finance, where transparency is crucial. Explainable AI (XAI) techniques, including LIME and SHAP, are increasingly being utilized to tackle this issue; however, they still fall short when it comes to capturing the decision-making logic of highly complex models (Heng and Subramanian, 2022). Additionally, problems related to data imbalance, especially the underrepresentation of default cases, highlight the need for techniques like SMOTE, ensemble oversampling, and adaptive weighting to achieve fair and accurate classifications (Jukna, 2022; Liang et al., 2022).

Ethical considerations are another significant theme, with many studies underscoring the potential for algorithmic bias, privacy violations, and misuse of sensitive financial or personal data (Heng and Subramanian, 2022). This necessitates the development of standardized evaluation protocols and ethical guidelines for deploying LLMs in risk-sensitive domains (Sarfati et al., 2024).

## 6 Conclusion

This review offers a thorough exploration of how NLP and Large Language Models are transforming the field of credit risk classification. By facilitating the integration of unstructured textual data and enhancing the semantic interpretation of financial narratives, models such as BERT, RoBERTa, and LLaMA represent a significant shift in risk modeling practices (Devlin et al., 2018; Liu et al., 2019; Touvron et al., 2023). These advancements not only enhance predictive accuracy but also introduce new avenues for analysis, including psychometric profiling, sentiment trajectory modeling, and multimodal fraud detection (Jin and Zhang, 2024; Yang et al., 2022; Xing, 2024; Che et al., 2024).

The integration of NLP into credit risk modeling presents several challenges. Key concerns regarding interpretability, fairness, regulatory compliance, and scalability continue to dominate discussions in both academic and industrial circles (Liu et al., 2020; Pathak et al., 2023; Heng and Subramanian, 2022). There is an evident necessity for more transparent models that can earn the trust of regulators and end-users alike. Additionally, it is crucial to address the prevention of existing biases and to safeguard user privacy within these models (Hassija et al., 2020; Boyapati and Aygun, 2025). The incorporation of NLP into credit risk modeling brings forth a number of challenges. Prominent issues related to interpretability, fairness, regulatory compliance, and scalability remain central to discussions in both academic and industry settings. There is a clear need for more transparent models that can foster trust among regulators and end-users alike. Moreover, it is essential to tackle the prevention of existing biases and ensure equitable outcomes.

In summary, although NLP and LLMs present significant opportunities for enhancing credit risk assessment, fully harnessing their advantages necessitates a balanced strategy that combines technical innovation with ethical responsibility (Lessmann et al., 2015; Heng and Subramanian, 2022; Sarfati et al., 2024). The future of credit risk modeling rests on the responsible and transparent application of these transformative technologies.

## Data Availability

The original contributions presented in the study are included in the article/supplementary material, further inquiries can be directed to the corresponding authors.
